# Guilt-inducing interaction with others modulates subsequent attentional orienting via their gaze

**DOI:** 10.1038/s41598-023-32283-3

**Published:** 2023-04-01

**Authors:** Wen Zhao, Jiajia Yang, Zhonghua Hu

**Affiliations:** Institute of Brain and Psychological Sciences, Sichuan Normal University, Chengdu, 610068 People’s Republic of China

**Keywords:** Human behaviour, Emotion

## Abstract

Gaze direction can trigger social attentional orientation, characterised by a speeded reaction time in detecting targets appearing in a gazed-at location compared with those appearing in other locations. This is called the ‘gaze-cueing effect’ (GCE). Here, we investigated whether a feeling of guilt established through prior interaction with a cueing face could modulate the gaze-cueing effect. Participants first completed a guilt-induction task using a modified dot-estimation paradigm to associate the feeling of guilt with a specific face, after which the face that had established the binding relationship was used as the stimulus in a gaze-cueing task. The results showed that guilt-directed faces and control faces induce equal magnitudes of gaze-cueing effect in 200 ms of stimulus onset asynchrony (SOA), while guilt-directed faces induce a smaller gaze-cueing effect than control faces in 700 ms SOA. These findings provide preliminary evidence that guilt may modulate social attention triggered by eye gaze at a later stage of processing but not in the earlier stages.

## Introduction

Gaze direction conveys a wealth of personal information, allowing one to quickly follow other people’s current focus of attention and infer their intentions as well as their mental states^1–3^. The ability to utilise information on gaze direction from social partners to deploy our attention resources accordingly is considered an essential skill in social interaction^4–6^.

It has been shown that observers tend to automatically follow the gaze of others, and this has been investigated in previous studies by a modified Posner paradigm, that is, the gaze-cueing paradigm^3,7–9^. During this type of task, a face with a direct gaze is presented in the centre of a screen, and it then looks to the left or right side of the screen. After a short variable stimulus onset asynchrony (SOA), a peripheral target randomly emerges on the left or right side of the screen. Participants are required to detect the target as quickly and accurately as possible. Although the gaze direction of the central face is neither predictive nor counter-predictive of the target location, participants’ reaction times (RTs) to targets at the gazed-at location are shorter than to targets at the non-gazed-at location^7,9–11^. This social phenomenon is termed the ‘gaze-cueing effect’ (GCE).

More recent studies have found that the GCE is modulated by social factors^12^, such as political affiliation^13,14^, social exclusion^15^, competitiveness^16^ and trustworthiness^17^. For example, Capellini^15^ used a *Cyberball* game—a brief ostracism-inducing manipulation wherein participants are left out of a three-way ball tossing game with two ostensible other participants who are in fact operated by a computer—to make participants feel excluded before performing a gaze-cueing task. They found that participants who felt excluded in the prior task exhibited a reduced GCE compared with those who were included. Furthermore, one study showed that social isolation experienced during the COVID-19 lockdown can increase GCE^18^. Moreover, faces associated with competitive relationships (i.e., a hostile relationship in a modified version of the two-choice *Prisoner’s Dilemma* game) trigger a stronger GCE relative to faces associated with cooperative relationships (i.e., a mutually beneficial relationship with the participant)^16^. Dalmaso^4^ suggested that faces that had engaged in a disjointed gaze with participants in a previous task elicited a GCE relative to those engaged in a disjointed gaze. The findings of their study showed that information from previous interactions could influence later gaze-following behaviours with the same people. The relationship between the observer and the cue face can modulate the GCE, suggesting that social interaction with others influences how their gaze shifts our attention.

Guilt, a negative experience-induced emotion, and responsibility for action in the aftermath of one person injuring another or violating moral principles^19^, is a social emotion that arises in and regulates social interactions^20^. In an interpersonal context, we feel guilty when our actions cause loss to be inflicted upon others^21^. As a moral emotion, guilt is related to understanding a victim’s thoughts, feelings and attitudes towards transgressors^21,22^. The feeling of guilt causes individuals to consider the concerns of others and increases reparative intentions, which help to enhance social relationships^23^. However, regarding guilty emotion and eye gaze cues, as eye contact may trigger feelings of being judged and accused for those who feel guilty, transgressors avoid gazing at the victim to reduce the resulting negative emotions^24,25^. Yu et al.^25^ found that participants fixated less on a partner’s eyes in a high-guilt condition in which participants had caused the partner’s pain than in a control condition in which the partner had caused pain. In this experiment, the arousal of guilt measured by skin conductance response was influenced by whether or not the victim’s eyes were seen. It has been demonstrated that interpersonal guilt is processed as a social threat^26^. After experiencing guilt, making eye contact with the victim induces anxiety^25^. Thus, participants who feel guilty are less likely to be influenced by the victim’s eyes. Moreover, previous research has found that the superior temporal sulcus region, which is known to play a prominent role in gaze cueing^27,28^, is also involved in the processing of guilt^29^. The same brain structure involved in processing the gaze cues and guilt suggests that some relationship between guilt processing and gaze processing may be reasonable. Additional evidence from neuroimaging studies suggests that the processing of guilt engages the same brain regions involved in social cognition and theory of mind, that is, the prefrontal and temporal regions^29–31^. Thus, guilt may have a significant effect on the perception of gaze direction and gaze-oriented attention.

The present study investigated whether the GCE is modulated by a guilt-directed face in a prior interaction. In the first task, we employed a dot-estimation task to induce guilt and manipulate the relationship between the participants and the cueing faces, with one face for the guilt condition (the participant was rewarded, while their partner lost the reward due to the poor performance of participants) and the other for the control condition (the participant and their partner were both rewarded). This method of inducing guilt has been employed in previous studies^21,22,32,33^. The faces were then used as the central face cue in a subsequent gaze-cueing task. Given that previous research has found that guilt could cause individuals to avoid eye contact with a victim. In addition, guilt is a directional emotion, which not only reflects the transgressor’s emotional state, but more importantly reflects the relationship between the transgressor and the victim. Thus, we expected that guilt would not have a general effect on GCE triggered by all faces cue, but only on GCE triggered by victim face. Participants may follow the gaze shift less in the guilt condition compared with the control condition. In other words, participants might exhibit a reduced GCE when the cues were victims’ faces rather than control faces. Furthermore, we used both 200 ms and 700 ms stimulus onset asynchrony (SOA), which is consistent with previous studies^34,35^, to explore the time course of the modulation induced by guilt on GCE. Some studies have shown that social modulation on GCE appears under long SOA conditions (e.g., facial expression^36^), while other studies have reported that the modulatory effect social factors on GCE emerges mainly under short SOA conditions (e.g., social status^37^). Taken together, the convergent evidence showed that SOA is a key factor in the modulation of social factors on GCE. Thus, it is plausible that the patterns in the modulation of guilt on GCE will vary across different SOAs.

## Methods

### Participants

Thirty-seven Chinese students (16 men and 21 women) aged between 18 and 22 years (mean age = 19.49 years, *SD* = 1.07 years) from Sichuan Normal University (China) participated in this study. A priori power analysis (see Supplemental Material for more details) using G*Power (Version 3.1.9.7^38^) indicated that a sample size of 34 participants was sufficient to detect a medium-sized main effect^15^ of eye gaze cues (f = 0.25) with α set at 0.05 and a power of 0.8. The sample size in previous studies investigating gaze-cueing effect or guilt emotion^10,21,32–34^ was involved between 20 and 55 participants, so we increased the sample size slightly to 37, which is about halfway between 20 and 55, to detect potential interaction in this study. All participants had normal or corrected-to-normal vision and were unaware of the purpose of the experiments. They all gave written informed consent to formally confirm their willingness to participate in the follow-up experiment. The experimental protocol was approved by the ethics committee of Sichuan Normal University [SCNU-201102]. All procedures were in compliance with the Code of Ethics of the World Medical Association (Declaration of Helsinki).

### Stimuli

Four neutral faces (2 men and 2 women) with three gaze directions (direct gaze and left- and right-averted gaze) were used in the experiment. For each participant, the two faces that were the same sex as the participant were randomly assigned to act as either the guilt-directed face or the control face. In the guilt-induction task, faces with a direct gaze (5.71° × 7.41°) were used to represent simulated players who were always of the same sex as the participant. In the gaze-cueing task, a white asterisk (0.48°) was used as the target, with images of faces (6.64° × 8.57°) gazing left or right. All stimuli were presented on a grey background, and participants sat approximately 60 cm from the screen. Stimulus presentation and response registration were controlled using MATLAB (Mathworks, Inc., Natick, MA, United States) software with Psychtoolbox extensions, and the order of conditions was counterbalanced across participants.

### Procedure

Participants were recruited online and came to the laboratory individually. A day before the experiment, participants were required to provide a profile photo with a neutral expression (the instruction was: “Please provide an electronic proof photo for formal experiments—not for any other purpose”), which would be presented on the screen as a representative of the participant in the feedback during the guilt induction task. On arrival, they were told that there were two experimental tasks, the first of which they would play with two partners sequentially and the other that they would finish by themselves.

### Face-specific guilt-induction task

**Figure 1:**
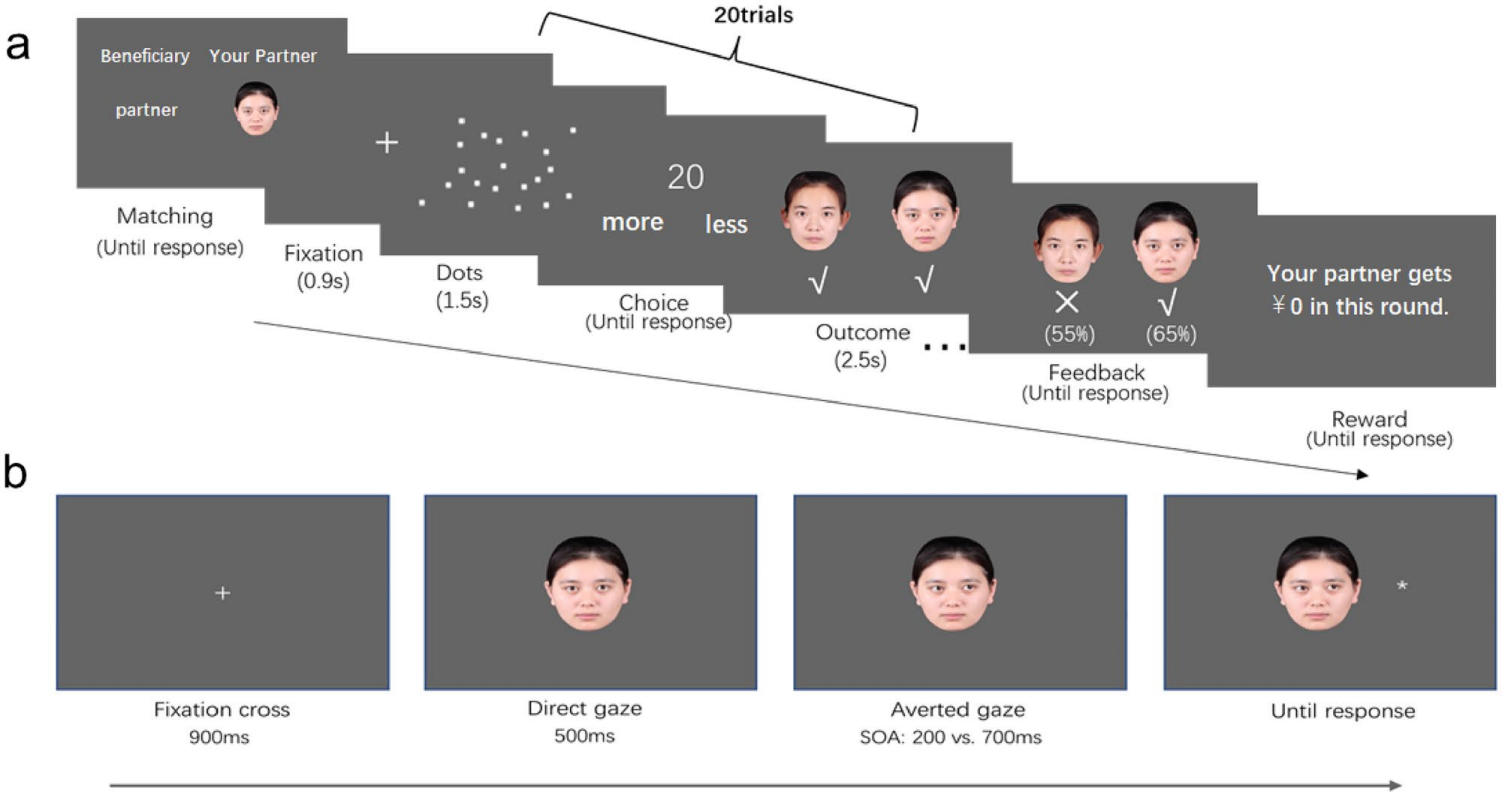
Experiment procedure. Upper panel (**a**): the face-specific guilt-induction task—an example of the guilt condition is depicted. At the beginning of each round, a partner, indicated by a face, and the bonus recipient, indicated by words, were shown. During each trial, images of the participant and the partner’s faces were presented on the outcome and feedback screen. The text in the screenshots of face-specific guilt-induction task was originally Chinese (see Supplemental Material for details). Lower panel (**b**): the gaze-cueing task—sample trials using a congruent condition example. The owners of example facial images consented to their portraits being published in any academic journal. Images were photograghs of four Chinese models and standardized with Photoshop software (version 22.5.8, www.adobe.com/products/photoshop).

In the face-specific guilt-induction task (see Supplemental Material for details), participants were instructed to play a dot-estimation task, ostensibly with two partners of the same gender as themselves (represented by two faces, which were randomly assigned to either guilt or control conditions) sequentially via a computer. At the beginning of each trial, a central fixation cross, “+” (0.5°) was presented first and lasted for 900 ms. Then, 20 white dots (the positions of the dots were randomly generated) were displayed on a screen with a grey background for 1500 ms. Next, one number (19 or 20) and two words (“more” and “less”) were displayed on the screen. The participants were required to quickly estimate the number of dots on the screen and determine whether there were more or less than 19 or 20 (these two numbers were random across trials). After responding, feedback for each trial was shown under the images for 2500 ms. In reality, the correctness of the estimation was predetermined by our experimental design. After one round containing 20 trials, a total correctness rate was given, followed by feedback on total bonuses (see Fig. 1a). To fully assess changes in participants’ emotional states induced by the experimental manipulation, after playing with a partner, participants were instructed to rate six emotions (guilt, shame, sadness, anger, happiness and pride) rather than just guilt on 7-point scales (1 = *not at all* to 7 = *very strongly*), which was a setup with reference to previous studies^21–23,32,33^.

Each participant played two rounds with one partner, totally played four rounds. Participants were told that if the correct rates of the two players were both higher than 60%, one player, who was designated as the beneficiary prior to each round, would get a bonus (i.e.￥10); otherwise, the player would get no bonus (i.e.￥0). In both the control and guilt conditions, the participant was the beneficiary of the bonus in the first round, and the ostensible partner was the beneficiary in the second round. In the control condition, both the participant and the partner earned the bonus due to their good performance in two rounds (in the gaze-cueing task, this partner’s face was defined as the control face). In the guilt condition, the participant earned the bonus in the first round due to the good performance of both the participant and the partner, but in the second round, the ostensible partner did not earn the bonus due to the participant’s poor performance (in the gaze-cueing task, this partner’s face was defined as a guilt-directed face). The order of conditions was counterbalanced across participants.

### Gaze-cueing task

In the gaze-cueing task (see Supplemental Material for details), a white fixation cross was presented on the screen for 900 ms, followed by a central face with a direct gaze for 500 ms. Then, depending on the SOA, the same face with a gaze averted rightward or leftward was presented for 200 ms or 700 ms^34^. After that, an asterisk (0.48° × 0.48°) was presented as a target on the left or right side of the screen until a response was made (see Fig. 1b). Participants were informed that the direction of gaze of the face was not informative and were instructed to identify the target location as quickly and accurately as possible with no requirement to maintain central fixation. If the target appeared on the left, participants were asked to press “1” with their right index finger on the numeric keypad of a full-sized keyboard; if it appeared on the right, participants were asked to press “4” with their right middle finger. Each participant completed 448 trials, preceded by 16 practice trials. The trial order was randomized with 50% congruent and 50% incongruent trials in the control as well as the guilt condition.

### Data analysis

All t-test and repeated-measures analysis of variance were performed using SPSS 18 software. Paired sample t-tests were conducted for the follow-up pairwise comparison. All follow-up pairwise comparisons were Bonferroni corrected. Cohen’s *d* and ${\eta }_{p}^{2}$ were used to estimate the effect size. We also reported the Bayes Factor (*BF*_10_), which was estimated by JASP software (version 0.11.1, JASP Team, 2019, jasp-stats.org) to provide ground for the magnitude of support for the alternative hypothesis over null hypothesis. Besides, Bayes Factors range from 0 to ∞, and a Bayes factor above 1 (specifically, 1 to 3 means a weak degree, 3 to 10 means a moderate degree, and 10 or more means a strong degree) indicates the stronger the degree to which the alternative hypothesis predicts the data than vice versa^39^.

In the analyses of RTs, anticipations (RTs < 100 ms) and delays (RTs > 3000 ms) were defined as outliers (0.09% of trials) and excluded from analysis. Errors (0.85% of trials) and further outliers (defined as RTs three *SD* above or below a participant’s mean 1.75% of trials) were excluded from the analysis. Previous studies have shown differences in the processing of gaze direction according to the sex of the participant^40–44^. Thus, in order to explore the possible sex differences in this study, participants’ sex was added into the repeated-measures analysis of variance as a variable. However, we did not find a significant main effect of participant sex and interaction with any other factors, *F*s < 1, *p*s > 0.1 (see Supplemental Material for details), so participant sex was not included in the analysis.

## Results

### Manipulation check

In the guilt condition, the ratings of guilt were significantly higher than the ratings of other emotions, *t*s (36) > 2.78, *p*s < 0.01, Cohen’s *d*s > 0.458, all BF_10_ > 4.888. Participants reported higher levels of guilt towards the face used in the guilt condition than in the control condition, *t* (36) = 7.04, *p* < 0.001, Cohen’s *d* = 1.158, BF_10_ > 1000, indicating that our manipulation was successful (see Table 1). In the control condition, the ratings of happiness and pride were significantly higher than the ratings of these emotions in the guilt condition, *t*s (36) <  − 4.61, *p*s < 0.001, Cohen’s *d*s <  − 0.757, all BF_10_ > 100, and the ratings of shame and sadness were higher in the guilt condition than the control condition, *t*s (36) > 2.64, *p*s < 0.05, Cohen’s *d*s > 0.434, all BF_10_ > 3.542.

**Table 1:** Results (M ± SD) of the manipulation check for the face-specific guilt-induction task.

Emotion	Guilt	Control	*t* (36)	*p*	Cohen’s *d*	BF_10_
Guilt	3.43 ± 1.64	1.97 ± 1.09	7.04	< 0.001	1.158	> 1000
Shame	2.43 ± 1.30	1.78 ± 1.18	2.64	0.012	0.434	3.542
Anger	2.30 ± 1.70	1.81 ± 1.24	1.92	0.062	0.316	0.925
Pride	2.24 ± 1.28	3.49 ± 1.48	− 4.87	< 0.001	− 0.801	973.704
Sadness	2.43 ± 1.21	1.51 ± 0.96	3.88	< 0.001	0.638	66.565
Happiness	2.41 ± 1.52	3.68 ± 1.68	− 4.61	< 0.001	− 0.757	468.357

### Reaction times

A 2 (face type: guilt-directed faces vs control faces) × 2 (congruency: congruent vs incongruent) × 2 (SOA: 200 ms vs 700 ms) repeated-measures analysis of variance (ANOVA) of mean RTs (for each participant, the mean RT in each experimental condition was calculated and used in analyses) revealed a significant main effect of congruency, *F*(1, 36) = 20.762, *p* < 0.001, ${\eta }_{p}^{2}$ = 0.366, due to shorter RTs in gaze-congruent trials (467.35 ± 83.39, *M* ± *SD*) than in gaze-incongruent trials (480.50 ± 79.85), as well as the main effect of SOA, *F*(1, 36) = 240.052, *p* < 0.001, ${\eta }_{p}^{2}$ = 0.870, due to shorter RTs at 700 ms SOA (448.02 ± 77.87) than at 200 ms SOA (499.83 ± 85.55). The main effect of face type was not significant *F*(1, 36) = 0.005, *p* = 0.946. The SOA × congruency interaction was significant, *F*(1, 36) = 7.326, *p* = 0.010, ${\eta }_{p}^{2}$ = 0.169. Follow-up pairwise comparisons showed that the RTs on gaze-incongruent trials were significantly longer than that on gaze-congruent trials at 200 ms SOA, *t*(36) = 5.241, *p* < 0.001, Cohen’s *d* = 0.810, BF_10_ > 1000, but not significant at 700 ms SOA, *t*(36) = 1.819, *p* = 0.059, Cohen’s *d* = 0.321, BF_10_ = 0.972.

**Figure 2:**
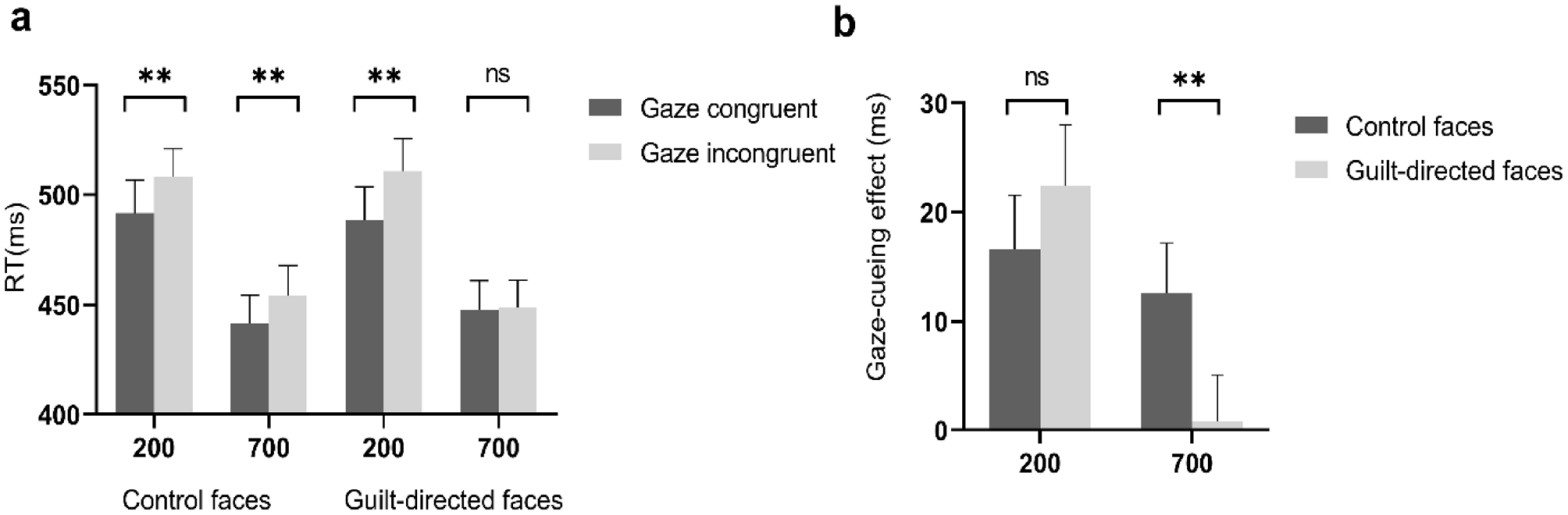
Interactions between face type, SOA and gaze congruency. The left panel (**a**) shows the three-way interaction between face type, SOA and gaze congruency. The right panel (**b**) shows the two-way interaction between SOA and face type. RT = reaction time. Gaze-cueing effect = RT_incongruent gaze_ − RT_congruent gaze_). *p < 0.05, **p < 0.01, ***p < 0.001, *ns* non significance. Bars depict *M* ± *SE*.

Critically, the three-way interaction between face type, congruency and SOA was significant, *F*(1, 36) = 5.152, *p* = 0.029, ${\eta }_{p}^{2}$ = 0.125 (see Fig. 2a). Follow-up Bonferroni-corrected pairwise comparisons (see Table 2) revealed that, at 200 ms SOA, the RTs in gaze-incongruent trials were significantly longer than those in gaze-congruent trials for both guilt-directed faces,* t*(36) = 3.997, *p* < 0.001, Cohen’s *d* = 0.657. BF_10_ = 90.499, and control faces, *t*(36) = 3.360, *p* = 0.002, Cohen’s *d* = 0.552, BF_10_ = 18.062; at 700 ms SOA, the difference in RTs between congruent and incongruent trials was significant only for control faces, *t*(36) = 2.783, *p* = 0.009, Cohen’s *d* = 0.457, BF_10_ = 4.801, not for guilt-directed faces, *t*(36) = 0.220, *p* = 0.827, Cohen’s *d* = 0.036, BF_10_ = 0.181. Neither the SOA × face type interaction nor the face type × congruency interaction approached statistical significance, *Fs* < 1, *ps* > 0.751.

**Table 2:** Reaction times (M ± SD, ms) for gaze congruency, face type and SOA.

	Control faces		Guilt-directed faces	
	200	700	200	700
Incongruent	508.16 ± 78.50	454.16 ± 83.36	511.00 ± 88.26	448.66 ± 76.05
Congruent	491.55 ± 92.00	441.54 ± 78.22	488.59 ± 90.77	447.73 ± 80.63
GCE	16.62 ± 30.08	12.62 ± 27.58	22.42 ± 34.12	0.93 ± 25.55
*t*	3.360	2.783	3.997	0.22
*p*	0.002	0.009	< 0.001	0.827

GCE = RT_incongruent_ − RT_congruent_; The *p*-values show the results of the post hoc tests comparing RT differences between gaze-congruent and gaze-incongruent conditions.

To investigate whether the magnitude of GCE is modulated by face type and SOA, we further calculated the GCE (mean incongruent RTs minus mean congruent RTs) and conducted an ANOVA with face type and SOA. A significant interaction between face type and SOA was found, *F*(1, 36) = 5.23, *p* = 0.029, ${\eta }_{p}^{2}$ = 0.13 (see Fig. 2b). Follow-up Bonferroni-corrected pairwise comparisons showed that the GCE decreased greatly for guilt-directed faces at 700 ms SOA compared with control faces,* t*(36) =  − 2.179, *p* = 0.035, Cohen’s *d* =  − 0.361, BF_10_ = 1.488, but not at 200 ms SOA, *t*(36) = 0.829,* p* = 0.413, Cohen’s *d* = 0.136, BF_10_ = 0.243.

## Discussion

In the current study, we combined a face-specific guilt-induction task and a gaze-cueing paradigm to investigate whether the GCE is modulated by guilty emotion associated with a cueing face during a prior interaction. We found that a three-way interaction between guilt condition, gaze-congruency and SOA such that, at 700 ms but not 200 ms SOA, guilt condition modulated the gaze-cueing effect, and the magnitude of the GCE for guilt-directed faces was significantly smaller than that for control faces, as strongly evidenced by the Bayes factors. This was such that the gaze-cueing effect emerged at an equivalent magnitude irrespective of guilt condition at 200 ms SOA but only occurred in the control condition at 700 ms SOA.

There are several possible interpretations of the GCE doesn’t emerge for guilt-directed faces. One possibility is that the feeling of guilt reduces the social interaction value of victims’ faces. Although guilt is considered an adaptive emotion that helps transgressors to maintain and improve relationships by asking for forgiveness or by making amends^23,45^. In our task, we did not give participants any way to compensate; in this case, the participants probably believed that the victim would resent them and would not want to socialise with them. Thus, participants may have been selectively less responsive to the gaze of guilt-directed faces, resulting in the disappearance of the GCE. Another possibility may be that the guilty feeling motivates social avoidance. After experiencing guilt, participants tended to change their behaviours to alleviate negative feelings. In our experiment, participants’ poor performance led to their partner’s loss, and they did not have the chance to compensate for their behaviours. Consequently, it is possible that seeing the victim’s eyes reminded participants of their poor performance in the prior task, reduced self-evaluation and enhanced the experience of guilt. To avoid this negative emotional experience, individuals may decrease their attention to the victim’s gaze.

To further determine whether the absence of GCE for guilt directed faces at 700 ms SOA was due to slower attention orientation or faster attention disengagement in the guilt condition, we did a further analysis (see Supplemental Material for details). We found that, in the cue-target congruent trials, participants had a slightly slower response to following the gaze direction of guilt-directed faces (448 ms) compared with control faces (442 ms), whereas at cue-target incongruent trials, participants tended to respond slightly faster to the target location in the guilt-directed faces condition (449 ms) than in the control faces condition (454 ms). This finding suggests that the feeling of guilt impedes an individual’s attention to orient to the location cued by cueing faces and prompts their attention to disengage from the cued location. These results need to be interpreted cautiously because, at 700 ms SOA, we did not find any significant difference in response times between guilt-directed faces and control faces in either the cue-target congruent or the cue-target incongruent trials.

Similar to previous studies, the findings of this study suggest that social information about a face acquired through an interactive game or background knowledge could influence a subsequent GCE elicited by this cueing face^16,34,46^. For example, the social reliability of faces learned from prior tasks affects participants’ responses to the gaze cues of these faces^47^, which suggests that our gaze response to others’ gazes can be influenced by prior social interaction with others.

In addition, we found that the GCE was modulated by face type at long SOA condition, but not at short SOA condition, which suggests that the modulation of guilt occurred at a later stage of processing, rather than at an earlier stage. This finding is consistent with electrophysiological evidence reporting that the modulation of social context on gaze direction processing appears in late event-related potential (ERP) components (for a review^48^). This, however, contradicts previous findings that have reported that possible modulation of social factors on GCE is observed mostly at shorter SOAs (i.e., 200 ms^37,49^). The finding of this study showed that the gaze and guilt emotion require time to be integrated. In the early processing stage, social attention orienting triggered by eye gaze is automatic and involuntary, and unaffected by guilt, whereas during the later processing phase, guilt associated with specific faces top-down suppresses the attentional shift triggered by eye cues.

Notably, in addition to guilt, we also found significant differences between the guilt and control condition in the rating scores of shame, sadness, happiness and pride. This finding suggests that guilt is a complicated emotion^32,33^. Importantly, the results of this study showed the ratings of guilt were higher than those of other emotions in the guilt condition, and the rating differences of guilt between the guilt and control condition were larger than that of other emotions, indicating the GCE differences between the guilt and control condition are predominantly contributed by guilt. Nevertheless, we cannot rule out the contribution of emotions other than guilt. Future research could optimise the experimental design to distinguish the contributions of different emotions to the experimental effect.

This study has some limitations. Firstly, we did not consider the gender effect between the face cue and the participants as a variable; instead, we only involved same-sex peers. In some cases women can show a greater gaze cueing effect than men^40,41^, so gender could be of interest when running gaze cueing studies. Therefore, it is unclear whether the attention-shifting induced by the same guilt-directed face cue would differ between same-sex and opposite-sex individuals. Future research could investigate this aspect further. Secondly, we asked participants to self-report on their emotional states immediately after the guilt-induction task to check whether the operation was effective, as has been done in previous studies^22,50^. However, it is inevitable that introspection about one’s emotional state may affect the experimental results. Future studies should require participants to self-report their emotional states after completing all experimental tasks to exclude the effect of emotional state introspection. Thirdly, since only Chinese volunteers were employed in this experiment, it is unclear if persons of other races would reach the same conclusions. For instance, Caucasian participants may have a distinct experience due to cultural background modification. Given the predicted reduction in caring for outcomes experienced by peers, Caucasian participants with a more individualistic viewpoint may be less vulnerable to guilt induction modulation (especially with strangers)^51^. Therefore, future studies could use Caucasian participants to further explore the possible cultural differences in the modulation of guilt on GCE.

In summary, the present study has demonstrated that feelings of guilt arising from a prior social interaction can modulate GCE, an effect that was further modulated by different SOAs, suggesting that high-level social information learned from social interactions guides our social attention.

## Supplementary Information

Supplementary Information.

## Data Availability

The data, material and experimental programs of this study are available at https://data.mendeley.com/datasets/m585w9g6r6.
