# Supplementary material for: Guilt-inducing interaction with others modulates subsequent attentional orienting via their gaze
Source: Sci Rep. 2023 Apr 1;13:5348. doi: 10.1038/s41598-023-32283-3 (PMC10067001; doi:10.1038/s41598-023-32283-3)
Supplement: Supplementary file 1 — Supplementary Information. [file 41598_2023_32283_MOESM1_ESM.docx]

Supplementary information for

**Guilt-inducing interaction with others modulates subsequent attentional orienting via their gaze**

*Wen Zhao ^a^, Jiajia Yang ^a^ and Zhonghua Hu ^a*^*

1. Institute of Brain and Psychological Sciences, Sichuan Normal University, Chengdu, PR China

**Correspondence to:**

Zhonghua Hu, Ph.D. Institute of Brain and Psychological Sciences, Sichuan Normal University, Chengdu, 610068, PR China, E-mail: [huzhonghua2000@163.com](mailto:huzhonghua2000@163.com)

***More details for Face-Specific Guilt-Induction Task***

In the guilt induction task, we chose a criterion like 60% to designate the guilt condition since our points were provided in a short period and in diverse placements, making it difficult for individuals to judge whether they did it right or not. The proper or wrong rate was predetermined. In the guilt condition, the first round was 65% accurate for the subject and 70% correct for the partner (the subject was awarded), and the second round was 55% correct for the subject and 65% correct for the partner (the partner was not rewarded). In the control condition, the first round was 65% accurate for the subject and 70% correct for the partner (the subject was awarded), and the second round was 70% correct for the subject and 65% correct for the partner (the partner was rewarded).

***More details for priori power analysis***

The exact parameters of the priori power analysis were as follows: Test family: F-test, statistical test: ANOVA repeated measures, within factors, and specified f = 0.25, alpha = 0.05, power = 0.8, Number of groups =1, Number of measures = 2, corr among rep measures = 0.5, which eventually yielded that 34 participants were needed.

**Table S1** *Four-way ANOVA of sex, congruency, face type, and SOA*

| Variables | *F* | *p* |
| --- | --- | --- |
| sex | 1.058 | 0.311 |
| type | 0.001 | 0.972 |
| type * sex | 0.577 | 0.453 |
| soa | 233.134 | 0 |
| soa * sex | 0.28 | 0.6 |
| gaze | 19.207 | 0 |
| gaze * sex | 1.738 | 0.196 |
| type * soa | 0.096 | 0.758 |
| type * soa * sex | 2.69 | 0.11 |
| type * gaze | 0.283 | 0.598 |
| type * gaze * sex | 0.189 | 0.666 |
| soa * gaze | 7.015 | 0.012 |
| soa * gaze * sex | 0.001 | 0.975 |
| type * soa * gaze | 5.049 | 0.031 |
| type * soa * gaze * sex | 0.043 | 0.837 |

***Additional analysis of RTs***

There was no significant difference in RTs between guilt-directed faces and control faces under congruent conditions at 700 SOA, *t* = 1.455, *p* = 0.154, nor in the incongruent condition, *t* = -1.326, *p* = 0.193.
